# 218. Impact of Prior Zoster Vaccination on Cardiovascular, Dementia, and Mortality Outcomes Following Herpes Zoster Infection: A Matched Cohort Study

**DOI:** 10.1093/ofid/ofaf695.076

**Published:** 2026-01-11

**Authors:** Ali Dehghani, George Yendewa

**Affiliations:** Department of Medicine, Case Western Reserve University School of Medicine, Cleveland, OH; Department of Medicine, Case Western Reserve University School of Medicine, Cleveland, OH

## Abstract

**Background:**

Herpes zoster (HZ) reactivation triggers systemic inflammation, increasing risks of vascular and neurologic complications. While vaccination reduces shingles incidence, its effect on long-term outcomes after breakthrough infections is unclear. We assessed whether prior zoster vaccination reduces major adverse cardiovascular events (MACE—myocardial infarction, stroke, pulmonary embolism, sudden cardiac death), dementia, and all-cause mortality in older adults with HZ. Secondary outcomes included psychiatric morbidity (anxiety, depression, bipolar disorder, schizophrenia) and Parkinsonism.

**Figure:**
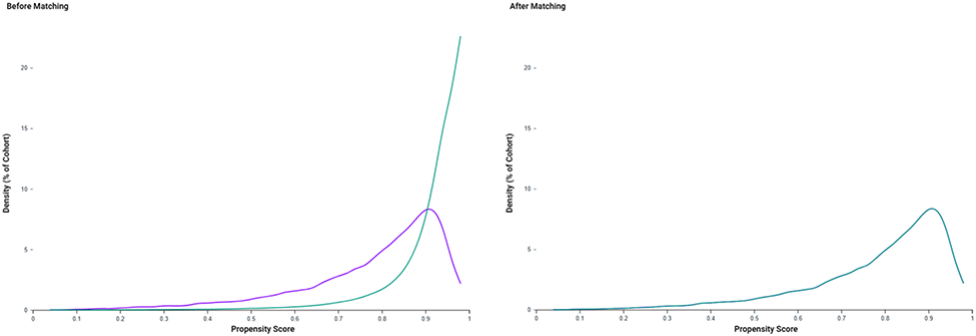
Propensity Score Density Before and After Matching: Zoster Vaccinated vs. Unvaccinated PLWH

Distribution of propensity scores in people living with HIV (PLWH) aged ≥50 years without prior HZ, comparing vaccinated (purple) and unvaccinated (teal) cohorts before (left) and after (right) 1:1 propensity score matching. Matching was based on demographics, ART regimen, cardiometabolic and psychiatric history, statin/antihypertensive use, and prior vaccine exposures. The post-matching plot demonstrates excellent overlap, confirming balanced covariate distribution between groups.

**Figure:**
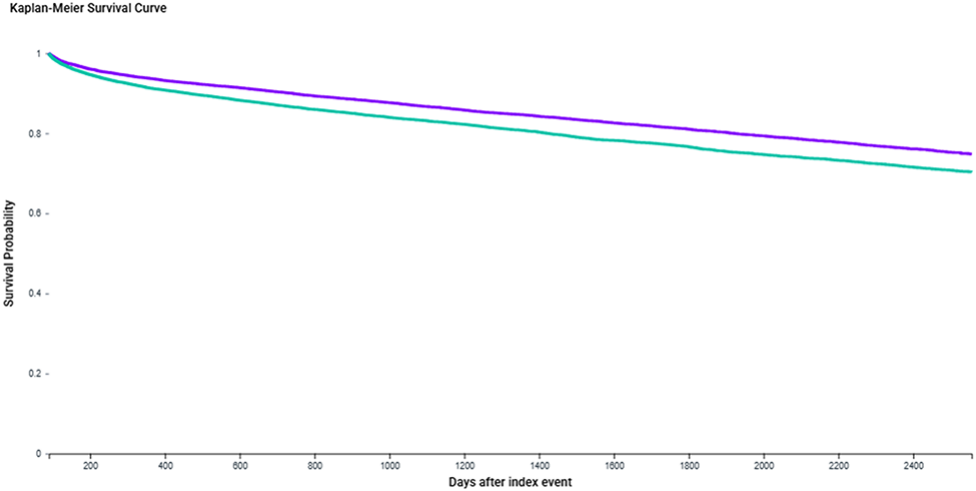
Kaplan-Meier Survival Curve for MACE in Vaccinated vs. Unvaccinated PLWH

Kaplan-Meier curve depicting time to first major adverse cardiovascular event (MACE) in people living with HIV (PLWH) aged ≥50 years without prior HZ, stratified by zoster vaccination status. The vaccinated cohort (purple) had significantly higher MACE-free survival compared to unvaccinated controls (teal) over a follow-up period of up to 7 years. Prior zoster vaccination was associated with a hazard ratio (HR) 0.787; 95% CI: 0.750–0.827; p=0.0001.

**Methods:**

We conducted a retrospective matched cohort study using the TriNetX Analytics Network. Adults aged ≥50 years with a first-time HZ diagnosis were stratified by prior zoster vaccination status: vaccinated (RZV or ZVL) versus unvaccinated (no record of zoster/varicella vaccination). Individuals with prior HZ, HIV, hepatitis B or C, ESRD, CNS infections, MACE, or dementia were excluded. Propensity score matching (1:1) was performed on demographics, comorbidities, psychiatric diagnoses, prior vaccine exposures, and chronic medications. Outcomes included MACE, dementia, and all-cause mortality, psychiatric morbidity and Parkinsonism. Hazard ratios (HRs) and Kaplan-Meier analyses were used to assess outcome differences, with p < 0.05 considered statistically significant.

**Figure:**
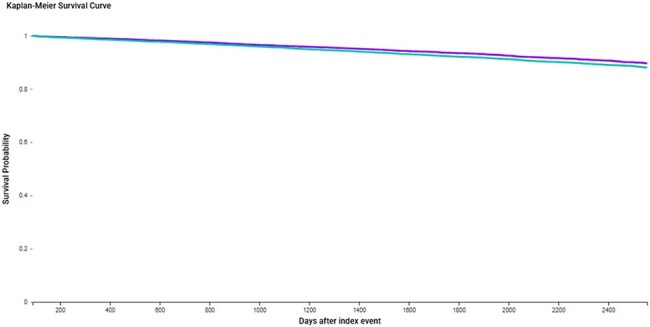
Kaplan-Meier Survival Curve for Dementia in Vaccinated vs. Unvaccinated Adults with Herpes Zoster

Kaplan-Meier curve comparing dementia-free survival between vaccinated (purple) and unvaccinated (teal) adults ≥50 years who developed a first-time herpes zoster (HZ) infection. Over a follow-up of up to 7 years, prior zoster vaccination was associated with a significantly lower hazard of dementia (HR 0.839, 95% CI: 0.772–0.913; p < 0.0001).

**Figure:**
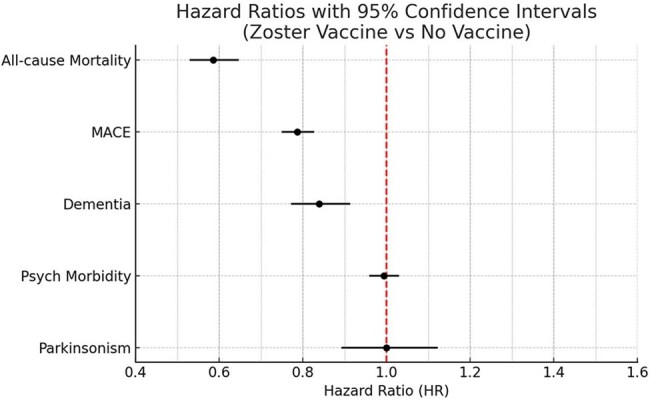
Forest Plot of Hazard Ratios for Clinical Outcomes: Zoster Vaccine vs. No Vaccine

Forest plot illustrating hazard ratios (HRs) with 95% confidence intervals for major outcomes in adults ≥50 years following herpes zoster (HZ) infection, comparing individuals with prior zoster vaccination to unvaccinated controls. Zoster vaccination was associated with significantly reduced risk of all-cause mortality (HR 0.586), major adverse cardiovascular events (MACE: HR 0.787), and dementia (HR 0.839), all with p < 0.0001. No significant differences were observed for psychiatric morbidity (HR 0.994, p = 0.7448) or Parkinsonism (HR 1.00, p = 0.9936).

**Results:**

Following 1:1 matching, A total of 38,092 patients (n=19,046 per group) were followed from 90 days to 7 years post-HZ (median follow-up was 3.6 years in the vaccinated group and 3.9 years in the unvaccinated group). The mean age was 69.1 years, 65% were female, and 69% were White. Prior zoster vaccination was associated with significantly lower hazards of all-cause mortality (HR 0.586, 95% CI: 0.530–0.647; p < 0.0001), MACE (HR 0.787, 95% CI: 0.750–0.827; p < 0.0001), and dementia (HR 0.839, 95% CI: 0.772–0.913; p < 0.0001). No statistically significant differences were observed in psychiatric morbidity (HR 0.994, 95% CI: 0.959–1.030; p = 0.7448) or Parkinsonism (HR 1.00, 95% CI: 0.892–1.122; p = 0.9936).

**Conclusion:**

Prior zoster vaccination reduced long-term risks of MACE, dementia, and mortality following HZ infection, supporting its role in mitigating post-viral complications.

**Disclosures:**

All Authors: No reported disclosures

